# Magnetorheological and Viscoelastic Behaviors in an Fe-Based Amorphous Magnetic Fluid

**DOI:** 10.3390/ma16051967

**Published:** 2023-02-28

**Authors:** Chuncheng Yang, Teng Li, Xiangyu Pei, Jiaxin Li, Zhao Yuan, Yan Li, Xiufang Bian

**Affiliations:** 1 Hebei Key Laboratory of Green Development of Rock and Mineral Materials, Hebei GEO University, Shijiazhuang 050031, China; 2 Institute of Basalt Fiber Materials, School of Gemmology and Materials Science, Hebei GEO University, Shijiazhuang 050031, China; 3 Engineering Research Center for Silicate Solid Waste Resource Utilization of Hebei Province, Shijiazhuang 050031, China; 4 Key Laboratory for Liquid-Solid Structural Evolution and Processing of Materials, Ministry of Education, Shandong University, Jinan 250061, China

**Keywords:** amorphous magnetic particle, magnetic fluid, rheological behavior, viscoelastic behavior

## Abstract

A novel magnetic fluid was obtained using a colloidal dispersion of amorphous magnetic Fe-Ni-B nanoparticles into water. Its magnetorheological and viscoelastic behaviors were all investigated. Results showed that the generated particles were spherical amorphous particles 12–15 nm in diameter. The saturation magnetization of Fe-based amorphous magnetic particles could reach 49.3 emu/g. The amorphous magnetic fluid exhibited shear shinning behavior under magnetic fields and showed strong magnetic responsiveness. The yield stress increased with the rising magnetic field strength. A crossover phenomenon was observed from the modulus strain curves due to the phase transition under applied magnetic fields. The storage modulus G′ was higher than the loss modulus G″ at low strains, while G′ was lower than G″ at high strains. The crossover points shifted to higher strains with increasing magnetic field. Furthermore, G′ decreased and fell off in a power law relationship when the strain exceeded a critical value. However, G″ showed a distinct maximum at a critical strain, and then decreased in a power law fashion. The magnetorheological and viscoelastic behaviors were found to be related to the structural formation and destruction in the magnetic fluids, which is a joint effect of magnetic fields and shear flows.

## 1. Introduction

Magnetic fluid (MF), also called ferrofluid, is a new type of intelligent material that is composed of magnetic nanoparticles, carrier liquid, and surfactants. MFs exhibit the fluid properties of non-magnetic liquid and the magnetic properties of solid magnetic materials. These unique properties of MFs make them attractive for a diverse range of applications, such as sealing [1], sensors [2], lubrication [3], heat transfer [4], and damping [5]. These applications are all strongly linked to the magnetorheological and viscoelastic behaviors of MFs, which are field controllable [6,7]. 

It is able to effectively control the MF flow by adjusting the direction and magnitude of magnetic fields. As the viscosities of MFs determine the working performance of the application, the viscosity changes induced by the magnetic field are of vital practical importance. The viscosity of an MF under a magnetic field was predicted by Rosensweig based on dimensional analysis and was shown to be a function of the ratio of hydrodynamic stress to magnetic stress [8]. Understanding the magnetoviscosity of magnetic colloids consisting of ferromagnetic particles was first proposed by McTague in 1969 [9]. Then, the magnetoviscous effect was systematically studied by Odenbach [10,11,12,13] and Zubarev [14,15,16]. Experiments indicated that some commercial MFs display clearly obvious magnetoviscous properties. The rise in viscosity under magnetic field can be one or two orders of magnitude, and strong magnetoviscous effects could be observed once the magnetic field is oriented along the velocity gradient of the MF flow [10,14]. It has been proven that such magnetorheological properties result from heterogeneous aggregates, which are chain-like and drop-like heterostructures [17,18]. Recently, the thixotropic behaviors of MF were systematically studied through hysteresis loop testing [19,20]. A meaningful hysteresis phenomenon was discovered from the viscosity–shear rate flow curves as a result of the thixotropy of the MF, which was caused by the existence and transformation of linear chain-like and drop-like structures [19]. The magnetoviscous effect of MF with various structures, surfactant concentrations, carrier liquids, and manufacturing technology was studied by Elena, et al. [21]. Their research demonstrated that the information on the magnetoviscous effect in a thin near-wall layer under conditions of shear oscillations holds promise for the lab on chip and microfluidic applications.

Because the magnetic particles in MFs accumulate together under applied magnetic fields and counterbalance the hydrodynamic forces, MFs demonstrate viscoelastic behaviors in rheological tests [22,23]. More broadly, the term “magnetoviscoelastic effect” was used to imply not only viscosity changes but also changes in the elastic properties because of the application of an external magnetic field [24]. The fundamental mechanisms of the change in the dynamic viscoelastic behaviors of ferrofluids were deduced as the connection and breakage of the (chain-like) side chains and (drop-like) dense clusters [23]. It was found that the temporal relaxation of the viscoelasticity and transport coefficient proves to be controlled by the intermediate scattering function of the colloidal suspensions [25]. In addition, a model theory of elasticity was proposed and verified by Polunin [26]. They found that the saturation magnetization of MF could be obtained by using the hydrostatic elasticity coefficient or ponderomotive elasticity coefficient if an MF was applied in a saturating magnetic field.

The magnetic properties of an MF is primarily assessed by the magnetic nanoparticles dispersed in MF. Fresh work has indicated that amorphous soft magnetic alloys, in particular Fe-based amorphous alloys, have extensive expectations for fabrication of MFs because of their unusual magnetic properties and low raw material cost. It has been shown that low coercivity and remanence properties can be improved in Fe-Co-B amorphous magnetic nanoparticles [27]. Fe-B, Fe-Ni-B, Co-B, and Co-Fe-Si-B amorphous particles have been synthesized and applied to MFs [28,29,30]. Moreover, amorphous Fe-Ni-B@OA magnetic nanoparticles have been able to enhance the oleophilic and hydrophobic properties of MFs [30]. Despite this, it is incredibly difficult to obtain amorphous nanoparticles by traditionally milled amorphous alloy ribbons because of high degrees of agglomeration. Moreover, amorphous MF is a new type of smart material, but research on the rheological and viscoelastic behaviors of Fe-based amorphous MFs is scarce and needs proper investigation.

In this study, Fe-Ni-B amorphous magnetic nanoparticles were prepared via a one-pot reaction, and oleic acid was used as a surfactant to modify the Fe-based amorphous magnetic particles. The structure, morphology, and magnetic properties of the particles were characterized. The magnetorheological and viscoelastic behaviors of Fe-based amorphous MFs were investigated. Our results indicate that the magnetic rheological and viscoelastic properties are induced by the combing effect of shear flows and magnetic fields, which is concerned with the evolution of the microstructures in the MFs. The present results provide a fresh point of view on the magnetorheological and viscoelastic behaviors of amorphous MFs.

## 2. Materials and Methods

Fe-based amorphous magnetic Fe-Ni-B particles were synthesized with ferrous sulfate, (FeSO_4_•7H_2_O), nickel chloride (NiCl_2_•6H_2_O), and sodium borohydride (NaBH_4_) by a one-pot reaction method. In this method, agar was directly added as the surfactant ahead of the chemical reduction process. All the chemical reagents utilized in the tests were of analytical reagent grade and used without additional purification.

**Figure 1:**
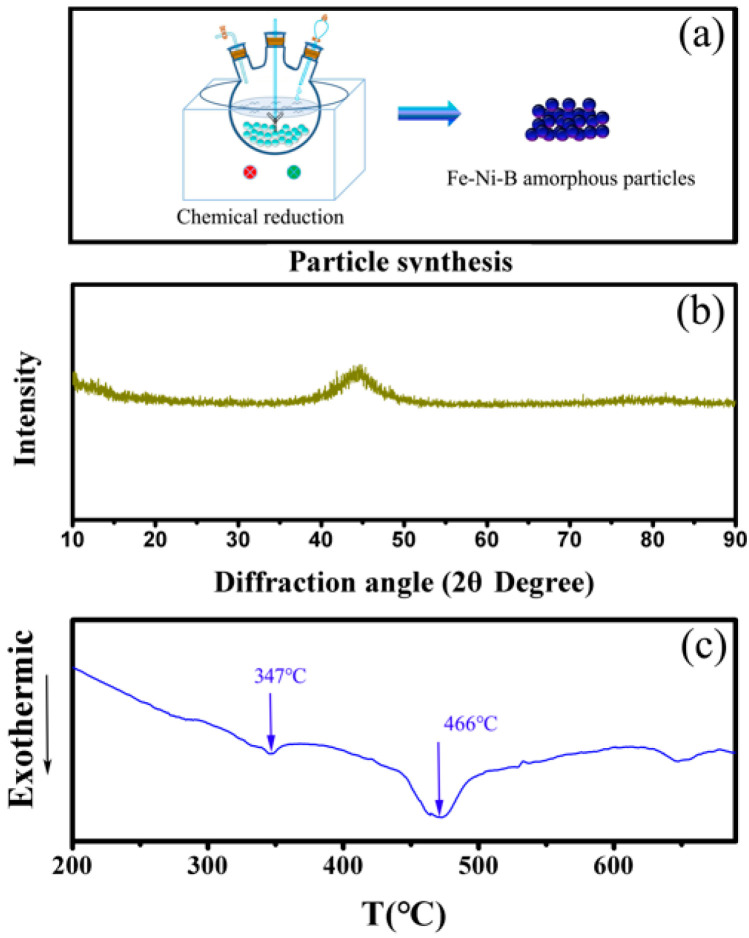
The sketch of Fe-Ni-B particle preparation (**a**); XRD patterns of Fe-Ni-B particles (**b**); DSC curves of Fe-Ni-B particles (**c**).

A solution was made by dissolving 11.9 g of nickel chloride (NiCl_2_•6H_2_O), 27.8 g of ferrous sulfate (FeSO_4_•7H_2_O), and 0.07g of agar into 100 mL of deionized water with mechanical stirring and supersonic dispersion together. The mixing solution was then stirred for 20 min until the blend was adequate. After that, the mixture was heated to 65 ℃. Next, 45 mL of 0.8 M NaBH_4_ aqueous solution was prepared and added into the mixture at a rate of 1.5 mL/min. NaOH solution was used to adjust the pH of the mixture to 11–12. After reacting for 30 min, the suspension was extracting using a centrifugal machine at high speed. The centrifugal products were then purified with water three times and cleaned with ethyl alcohol twice. Lastly, Fe-Ni-B amorphous magnetic nanoparticles modified with agar were obtained. The Fe-based amorphous MF was prepared using water as the base liquid. Appropriate amounts of amorphous Fe-Ni-B magnetic nanoparticles coated by agar were dispersed in water to prepare the MFs. Finally, an Fe-based amorphous MF with a volume fraction of 3% was obtained. The sketch of amorphous Fe-Ni-B magnetic particle preparation is shown in Figure 1a.

The structure of the Fe-Ni-B particles was characterized by X-ray diffraction (XRD, Netherlands PANalytical, EMPYREAN, Jinan, China) measurements with D/max-Rb and Cu Kα as radiation. The morphologies of amorphous Fe-Ni-B magnetic particles were identified by scanning electron microscopy (SEM, Germany ZEISS, SUPRA^TM^55, Jinan, China) and transmission electron microscopy (TEM, Japan, JEM-F200). The thermal performance of the amorphous Fe-Ni-B magnetic particles was investigated by differential scanning calorimetry (DSC, Germany, Netzsch DSC 404 C, Jinan, China) at a heating rate of 20 K/min from room temperature to 800 ℃. The magnetic properties of the particles and MF were studied by a superconducting quantum interference device (SQUID, USA Quantum Design, MPMS-3, Jinan, China) at room temperature. The hydrodynamic diameter of the particles in MF was determined by dynamic light scattering (DLS, MalvernPanalytical, Zetasizer, Jinan, China). An MCR 301 (Austria Anton Paar, MCR301, Beijing, China) rotational rheometer attached to a magneto rheological device (MRD170) was applied to investigate the rheological and viscoelastic properties of the Fe-based amorphous MFs. The MRD is incorporated in the rheometer’s measurement and control system. Moreover, an improved homogenous field distribution in the sample can be obtained using a magnetic yoke cover. Here, a cone–plate geometry was chosen for the measurement system, which is particularly produced with non-magnetic conduction material for magnetorheological measurements. This can make sure that the shear rate is constant in the sample. Moreover, it can also ensure that the influence of the shear on the cluster formation of magnetic particles is steady and uniform. The magnetic field in the measurement system is in the direction of the rotational axis of the cone–plate arrangement. Our experiments were conducted under relatively small field strengths of less than 0.3 T to obtain a relatively uniform magnetic field in the sample. In the present work, each test was repeated no less than three times. The measurement error in our experiments was less than 1%.

## 3. Results and Discussion

The XRD pattern of the Fe-Ni-B particles is demonstrated in Figure 1b. We can see that the XRD pattern of Fe-Ni-B particles displays a “steamed buns peak”, which is in the 2θ range of 35–55°. This result indicates that the Fe-Ni-B particles have amorphous structures. Moreover, no secondary peaks are exhibited, indicating that the obtained Fe-Ni-B amorphous particles are pure. The DSC curve of the amorphous particles is displayed in Figure 1c. Two exothermic peaks in the curve at 347 and 466 ℃ exist, indicating two steps in the crystallization process. Both the XRD and DSC results demonstrate that the Fe-Ni-B particles are amorphous.

**Figure 2:**
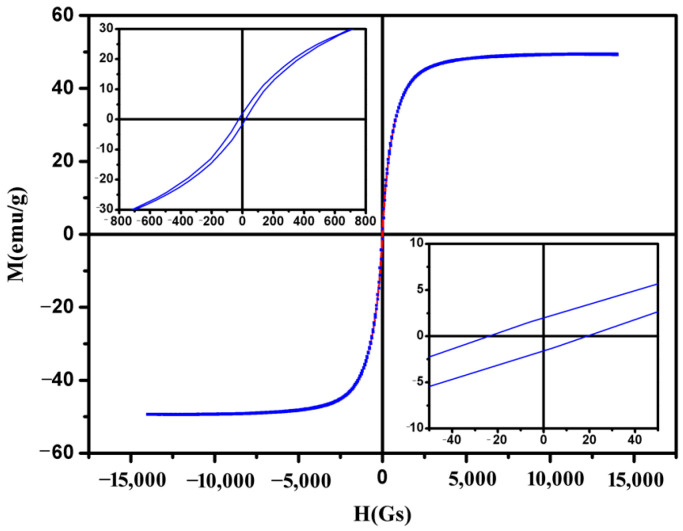
The magnetic hysteresis curves of amorphous magnetic Fe−Ni−B nanoparticles.

Figure 2 shows the magnetic hysteresis curves of the amorphous Fe-Ni-B magnetic particles. We can see that the Fe-Ni-B particles exhibit no hysteresis. Moreover, remanent magnetization and coercivity are very small. The values of magnetic remanence and magnetic coercivity are 2.4 emu/g and 23 Oe, which can almost be neglected (see the insert), indicating the superparamagnetism of the amorphous Fe-Ni-B magnetic particles. It can be seen that the saturation magnetization (*M_s_*) of the particles is about 49.3 emu/g. It is helpful for the recycling and fabrication of MFs if the particles exhibit a powerful magnetic response to the applied external magnetic field.

**Figure 3:**
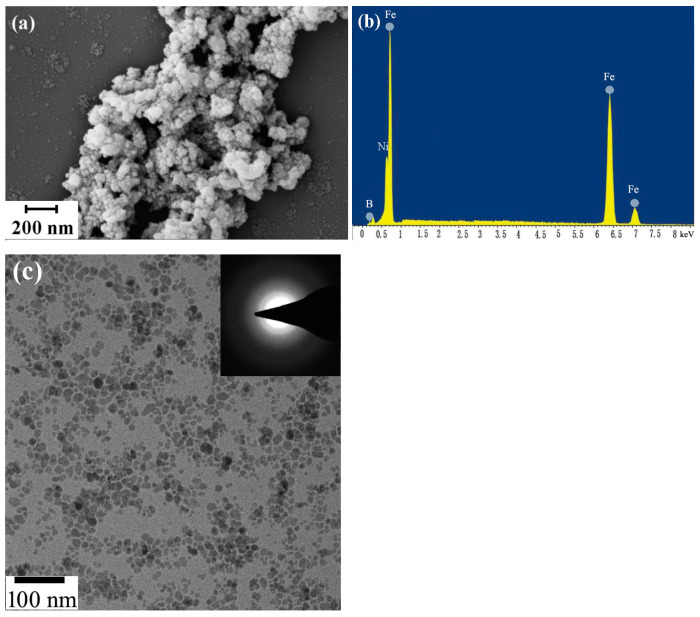
SEM image of amorphous Fe-Ni-B particles (**a**), EDX spectra of Fe-Ni-B amorphous magnetic particles (**b**), and TEM image of amorphous Fe-Ni-B particles (**c**).

A representative SEM image (Figure 3a), EDX spectra (Figure 3b), and a TEM image (Figure 3c) of the amorphous Fe-Ni-B magnetic particles are demonstrated in Figure 3. The mean particle size of amorphous Fe-Ni-B magnetic particles is about 12–15 nm. The Fe-Ni-B particles display slight aggregation because of their nanometer sizes as seen in the SEM image. The EDX result in Figure 3b shows that the elemental contents are Fe, Ni, and B. The Fe-Ni-B particles shown in Figure 3c are almost spherical with rough surfaces and demonstrate nearly no aggregation. The inserted selected area electron diffraction (SAED) image in Figure 3c exhibits a typical halo diffraction of an amorphous structure of the Fe-Ni-B magnetic particles. The result agrees with the XRD and DSC data mentioned above.

**Figure 4:**
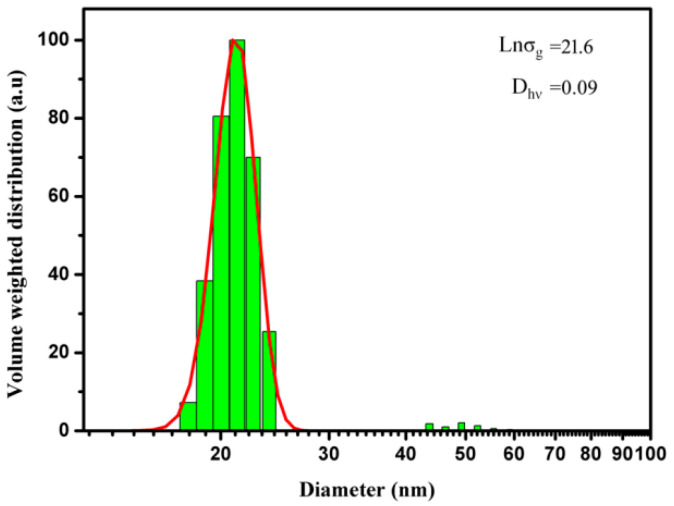
Hydrodynamic diameter of the Fe-Ni-B particles.

Figure 4 demonstrates the hydrodynamic diameter of the amorphous Fe-Ni-B magnetic nanoparticles. Samples for DLS tests were obtained via suspending dry amorphous magnetic particles in hexane. A hydrodynamic volume weighted diameter (*D_hv_*) of 21.6 nm with a geometric deviation Lnσ_g_ of 0.09 was obtained by fitting the volume weighted hydrodynamic diameter distribution. This result indicates that the nanoparticles exhibit narrow size distribution and disperse well in hexane, although there are small fractions of aggregates sized in the 43–55 nm range.

**Figure 5:**
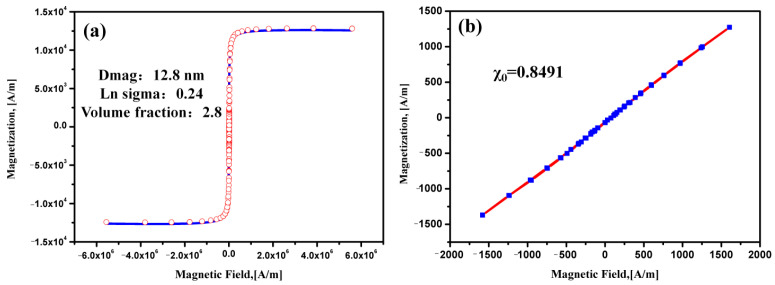
The magnetic hysteresis curves of the amorphous MF (**a**) and magnetization curve at low fields of the amorphous-based MF (**b**).

The magnetic hysteresis curve as well as a magnetization curve at low fields of the amorphous-based MFs are shown in Figure 5. The MF exhibits a superparamagnetism property with a *M_S_* of 12.6 kA/m. We obtained the magnetic particle volume fraction φ (φ = 2.8 ν/V) through fitting the Langevin function which is weighted by the lognormal size distribution. The magnetic core diameter *D_mag_* of the amorphous Fe-Ni-B particles in MFs is 12.8 nm with a geometric deviation of *Lnσ_g_* = 0.24. In addition, the magnetization curve at low fields of the amorphous MFs demonstrate that the χ_0_ is 0.8491, indicating the easy magnetization properties of the amorphous Fe-Ni-B MF.

**Figure 6:**
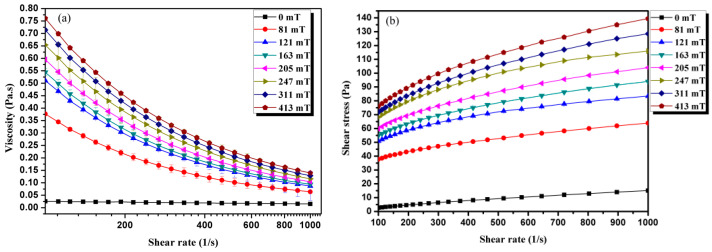
The viscosity of MF as a function of shear rates (**a**); shear stress vs. shear rate curves (**b**).

Viscosity vs. shear rate curves under various magnetic fields is displayed in Figure 6a. The viscosity of the Fe-based amorphous MF was measured at 25 ℃. The sudden change in the shear rate and magnetic field could induce the instantaneous effect. To avoid this, we conducted a pre-shearing measurement for 300 s at a shear rate of 11 s^−1^ without an applied magnetic field. Then, a shear rate of 5 s^−1^ for 10 s with a specified magnetic field was applied, which was then followed by a shear rate ramp range from 100 to 1000 s^−1^. It is noted that shear thinning phenomena were observed from the curves, where the viscosity decreased with increasing shear rate under different magnetic fields. This shear thinning behavior is associated with the microstructural change in the MF [31]. Initially, there were column-like structures in the MFs. The large column-like structures break into smaller ones or single chains with increasing shear rates, resulting in lowered viscosity of the MFs. The shear stress vs. shear rate curves are displayed in Figure 6b. It can be seen that the shear stress raised with the increase in magnetic field strength. The shear stress was shown to be dependent on the shear rate once a constant magnetic field was applied to the MF. These amorphous MFs exhibit characteristics similar to that of a Bingham plastic fluid.

**Figure 7:**
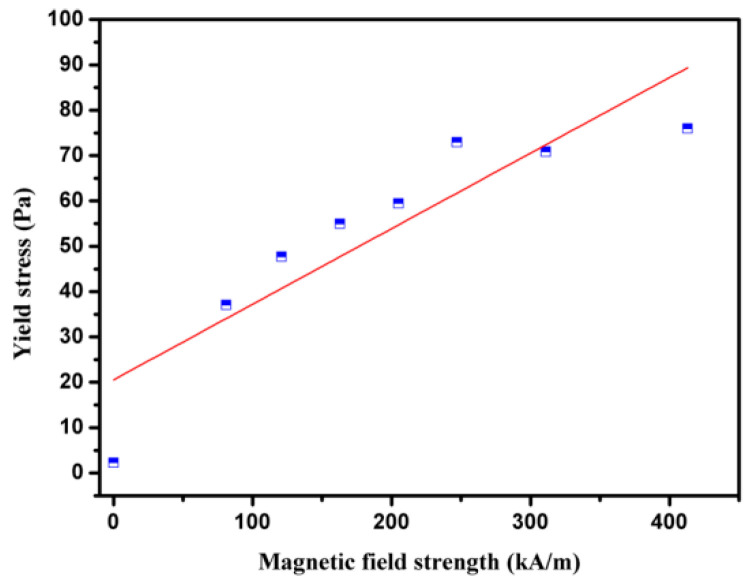
The yield stress of the amorphous MF as a function of magnetic field.

Yield stress is a crucial element in judging whether an MF is suitable, particularly for commercial uses. The yield stress of Fe-based amorphous MFs as a function of magnetic field is shown in Figure 7. It was found that an increasing magnetic field causes increases in yield stress. This is due to the fact that the field-induced chain-like or drop-like structures restrict the fluid more with increasing the magnetic field, which thereby improves the magnetorheological effects of the Fe-based amorphous MFs.

**Figure 8:**
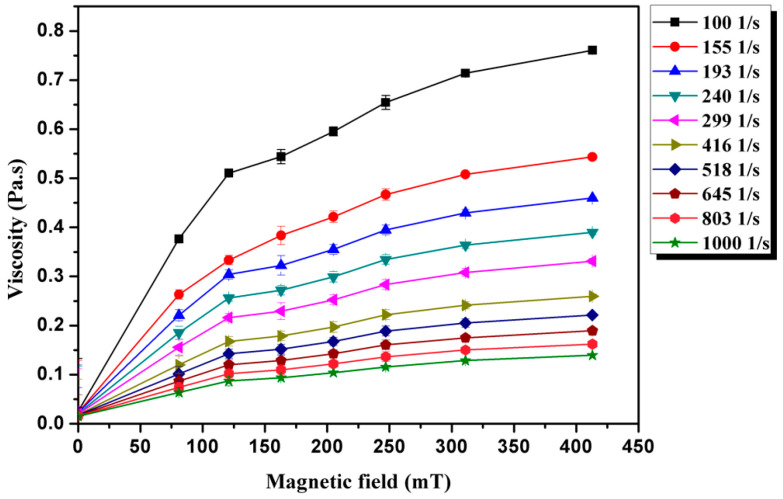
The viscosity of Fe-based amorphous MF as a function of magnetic field.

It is widely known that magnetic field has an obvious effect on the viscosity of an MF [9,10]. Generally, the viscosities of MFs increase with increasing magnetic field. The viscosity of Fe-based amorphous MFs as a function of magnetic field is shown in Figure 8. The viscosity of the MFs is established by the viscosity of the carrier liquid (water) and the interactions of the magnetic particles. It is a well-known that the viscosity of water is not influenced by the magnetic field, whereas the magnetic particles in the MF can become polarized by the magnetic field. Magnetic particles arrange their orientation along the direction of the magnetic field when a magnetic field is applied. The interaction and arrangement strengthen, and the flow resistance increases with increasing magnetic field. Therefore, the viscosity of the Fe-based amorphous MF increases under applied MFs. Research [15,16] shows that the magnetic field can generate aggregation and formation of chain-like or drop-like structures with increasing magnetic field strength, which leads to significantly increased viscosity of MFs. Laboratory and computer experimental investigations have demonstrated that the heterogeneous structures in MFs are linear chains and bulk drop-like structures in MFs. The presence of the chain-like and drop-like structures can clearly influence the rheological properties of Fe-based amorphous MFs. In addition, the dependence of viscosity on the magnetic field strength, in addition to the formation of chain structures, are a small gap in the rheometer d = 0.027 mm, the presence of aggregates in it, as well as the colloidal stability of the resulting magnetic fluid. As a result, several aggregates can close the gap in the rheometer, which has a strong effect on the viscosity. 

**Figure 9:**
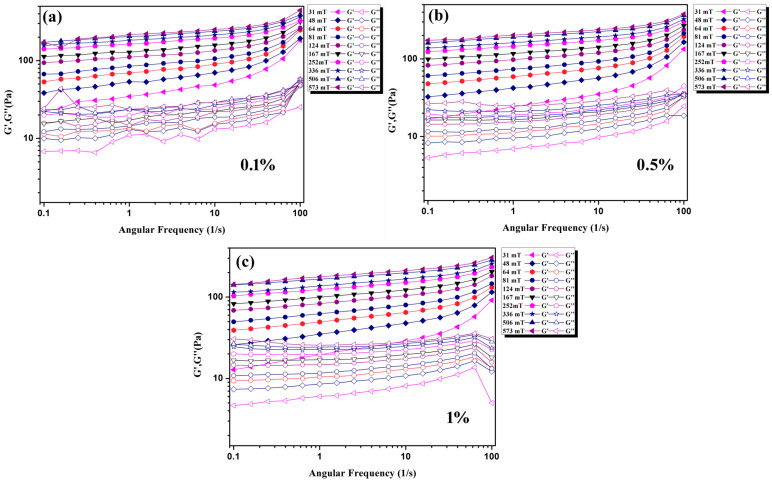
The storage modulus G′ and loss modulus G″ as a function of frequency at 0.1% (**a**), 0.5% (**b**), and 1% strain (**c**).

Dynamic oscillatory testing was conducted under angular frequency sweep. Storage modulus G′ and loss modulus G″ were characterized as a function of frequency at room temperature under angular frequency sweeps of 0.1–100 rad/s at 0.1%, 0.5%, and 1% strain. The results are shown in Figure 9. The storage modulus G′ is concerned with the capacity of the material to store energy as soon as an oscillatory force is applied and the loss modulus G″ is concerned the ability of the material to lose energy [32]. We can see that solid-like behavior (G′ > G″) was observed from the dynamic oscillatory responses. The results indicate that Fe-based amorphous MFs have a strong ability to store energy especially under applied MFs.

**Figure 10:**
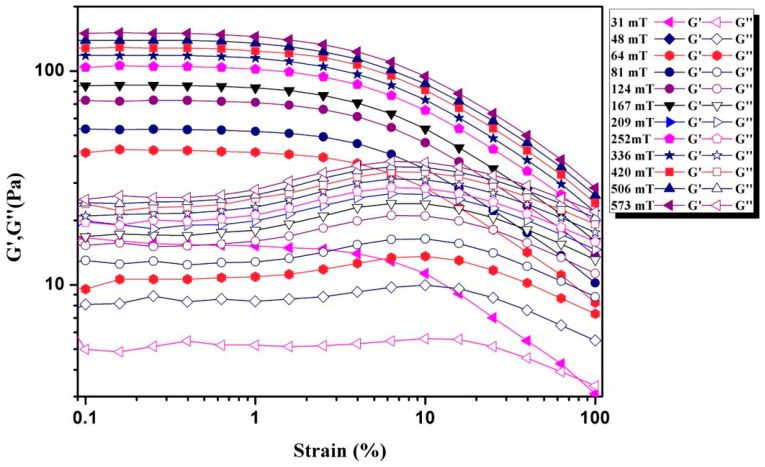
The variation in storage and loss moduli with respect to strain amplitude at an angular frequency of 10 rad/s.

Storage modulus G′ and loss modulus G″ were also measured to study the viscoelastic behavior of Fe-based amorphous MFs. The variation in storage and loss moduli with respect to strain amplitude at an angular frequency of 10 rad/s is shown in Figure 10. It can be seen that the storage modulus G′ is greater than the loss modulus G″ at a low strain. Then with increasing the strain, the storage modulus G′ decreases to less than the loss modulus G″ after a certain critical strain. Moreover, the critical strain point moves to a larger strain with rising magnetic field. The elastic effect is dominant over the viscous effect at low strain, resulting in higher storage modulus, thereby indicating the existence of stronger connections between the magnetic nanoparticles. In other words, the magnetostatic forces play a more prominent role than the hydrodynamic forces at this stage. However, the phenomenon reverses with increasing strain. The hydrodynamic forces became dominant over the magnetostatic forces because the viscosity effect of the fluid exceeded the elastic effect and the magneto viscoelastic effect became reduced. Moreover, the magnetic fields have obvious effect on the magneto viscoelastic effect of the MF, leading to the critical point shifting to higher strain.

With respect to structure, some key points include the following. Chain-like or drop-like structures were formed in the MFs with the applied magnetic field. The appearance of these structures results in an increase in storage modulus at low strain, where the polydispersity of the MFs plays a significant part. The biggest Fe-based amorphous magnetic particles form drop-like structures and determine the magnetic viscoelastic properties when the magnetic field strength is comparatively small at low strain. When the magnetic field strength rises, the relatively small Fe-based amorphous magnetic particles form new chain-like and drop-like structures, giving rise to further increases in storage modulus. According to this, it is easy to understand that the formation of chain-like and drop-like structures can appear with an applied magnetic field and affect the magnetic viscoelastic properties of the amorphous MFs. At a high strain rate, because of the hydrodynamic forces, the magnetic field-induced structures break, causing crossovers between loss modulus and storage modulus. Drop-like or chain-like structures were completely disrupted and amorphous magnetic particles were dispersed finely at high strains. The viscoelastic system becomes mastered by viscous behavior. The crossover occurring at a critical strain shifts to a higher strain with growing applied magnetic fields. The magnetic viscoelastic behavior accompanying the phase transition in the Fe-based amorphous MFs could make this kind of MF valuable for diverse industrial uses, especially advanced device applications.

The application of a magnetic field imparts a viscoelastic nature to MFs. It is well known that the storage modulus represents the elastic solid-like behavior and the loss modulus indicates the viscous response [33,34]. Here, in Figure 10, it is shown that the storage modulus is larger than the loss modulus in the presence of a magnetic field. In addition, both moduli keep constant as far as a certain strain value, indicating a predominant solid-like behavior of the MFs. As mentioned above, the innate character of elastic is imparted by the magnetic field. It causes the formation of chain-like and drop-like structures. Nevertheless, as the strain exceeds a critical value, the motion of the magnetic field-induced structures is no longer affine, resulting in particle rearrangements. The higher strain leads to a destruction of the magnetic field-induced structures. At values greater than the critical shear rate, the storage modulus decreases in a power law relationship. However, the loss modulus demonstrates a distinct maximum at a critical strain, and then reduces in a power law relationship, which is a normal feature of soft glassy materials. The viscoelastic system is associated with either efficiently-conserved or dissipated energy [35]. Because of the utmost dissipation of energy during the start of the destruction of chain- or drop-like structure, the loss modulus exhibit a peak at the critical strain seen in Figure 10. Our results are similar to the result in Ref [34], in which a soft glassy behavior in a magnetic colloid exposed to an external magnetic field was observed and discussed.

## 4. Conclusions

In this paper, a new kind of amorphous MF was prepared by dispersing Fe-based amorphous magnetic particles into water. The prepared magnetic spherical particles were prepared via chemical reduction and exhibited 12–15 nm diameters. The saturation magnetization of the obtained particles was 49.3 emu/g. The magnetorheological and magnetoviscoelastic properties of the amorphous MFs were studied. It was found that the MFs exhibited shearing thinning behavior and the yield stress increased with increasing magnetic field strength. The storage modulus G′ was greater than the loss modulus G″ under the applied magnetic field and both moduli kept constant at first. The storage modulus G′ reduced in a power law relationship when the strain surpassed a critical value. However, loss modulus G″ demonstrated a distinct maximum at a critical strain, and then decreased as a power law. The viscoelastic properties can be explained by the chain- or drop-like structure formation–destruction process, which is a competition between the interactions of magnetic particles in the MF and the shear flows. The present results can provide a new point of view on the application of MFs that also accounts for the viscoelastic effects.

## Data Availability

The data presented in this study are available on request from the corresponding author.

## References

[1] Yang X., Liu Y., Zhang R., Gao S. (2021). Experimental research on self-repairing of diverging stepped magnetic fluid seals with single magnetic source. J. Magn..

[2] Wang M., Jin W., Liu X., Sun W., Zhang C., Bi W. (2022). Design and research of magnetic field sensor based on grapefruit optical fiber filled with magnetic fluid. Optoelectron. Lett..

[3] Fan H., Zhou J., Wang Y. (2016). Design of Non-contact Mechanical Seal Lubricated by Magnetic Fluid. Lubr. Eng..

[4] Izadi M., Javanahram M., Zadeh S.M.H., Jing D. (2020). Hydrodynamic and heat transfer properties of magnetic fluid in porous medium considering nanoparticle shapes and magnetic field-dependent viscosity. Chin. J. Chem. Eng..

[5] Miriyala D., Goyal P.S. (2022). Effect of magnetic field on the damping behavior of a ferrofluid based damper. arXiv.

[6] Sedlacik M., Pavlinek V., Saha P., Svrcinova P., Filip P. (2011). The role of particles annealing temperature on magnetorheological effect. Mod. Phys. Lett. B.

[7] Sedlacik M., Moucka R., Kozakova Z., Kazantseva N.E., Pavlínek V., Kuritka I., Kaman O., Peer P. (2013). Correlation of structural and magnetic properties of Fe_3_O_4_ nanoparticles with their calorimetric and magnetorheological performance. J. Magn. Magn. Mater..

[8] Rosensweig R.E. (1985). Ferrohydrodynamics.

[9] McTague J.P. (1969). Magnetoviscosity of magnetic colloids. J. Chem. Phys..

[10] Odenbach S. (2002). Magnetoviscous Effects in Ferrofluids.

[11] Odenbach S., Raj K. (2000). The influence of large particles and agglomerates on the magnetoviscous effect in ferrofluids. Magnetohydrodynamics.

[12] Odenbach S. (2003). Ferrofluids-magnetically controlled suspensions. Colloids Surf. A.

[13] Fröhlich J., Schwarz S., Heitkam S., Santarelli C., Zhang C., Vogt T., Boden S., Andruszkiewicz A., Eckert K., Odenbach S. (2021). Influence of magnetic fields on the behavior of bubbles in liquid metals. Eur. Phys. J. ST (Spec. Top.).

[14] Zubarev A.Y., Odenbach S., Fleischer J. (2002). Rheological properties of dense ferrofluids: Effect of chain-like aggregates. J. Magn. Magn. Mater..

[15] Zubarev A.Y., Yu L. (2004). Chain-like structures in polydisperse ferrofluids. Phys. A.

[16] Zubarev A.Y., Yu L. (2004). To the theory of rheological properties of ferrofluids: Influence of drop-like aggregates. Phys. A.

[17] Ishida S., Yang Y., Meng F., Matsunaga D. (2021). Field-controlling patterns of sheared ferrofluid droplets. Phys. Fluids.

[18] Jain S.K., Banerjee U., Mandal C., Sen A.K. (2022). Reversible transition from ferrofluid drop to spikes due to an approaching magnet. Eur. Phys. J..

[19] Yang C., Liu Z., Yu M., Bian X. (2020). The influence of thixotropy on the magnetorheological property of oil-based ferrofluid. J. Mol. Liq..

[20] Li Z., Li D., Chen Y., Guo Y., Zhang Z. (2019). Thixotropic yielding behaviors of ferrofluids. J. Magn. Magn. Mater..

[21] Sheldeshova E., Churaev A., Ryapolov P. (2023). Dynamics of Magnetic Fluids and Bidisperse Magnetic Systems under Oscillatory Shear. Fluids.

[22] Li Z., Li D., Hao D., Cheng Y. (2017). Study on the creep and recovery behaviors of ferrofluids. Smart Mater. Struct..

[23] Felicia L.J., Philip J. (2014). Probing of field-induced structures and their dynamics in ferrofluids using sscillatory rheology. Langmuir.

[24] Melzner K., Odenbach S. (2002). Investigation of the Weissenberg effect in ferrofluids under microgravity conditions. J. Magn. Magn. Mater..

[25] Avdeev M., Balasoiu M., Torok G., Bica D., Rosta L., Aksenov V.L., Vekas L. (2002). SANS study of particle concentration influence on ferrofluid nanostructure. J. Magn. Magn. Mater..

[26] Polunin V.M., Ryapolov P.A., Platonov V.B., Sheldeshova E.V., Karpova G.V., Aref’ev I.M. (2017). Elasticity of a Magnetic Fluid in a Strong Magnetic Field. Phys. Acoust..

[27] Zhao S., Bian X., Yang C., Yu M., Wang T. (2018). Synthesis of FeCoB amorphous nanoparticles and application in ferrofluids. Appl. Surf. Sci..

[28] Wang T., Bian X., Yang C., Zhao S., Yu M. (2016). Ferrofluids based on Co-Fe-Si-B amorphous nanoparticles. Appl. Surf. Sci..

[29] Yang C., Liu Z., Yu M., Bian X. (2020). Liquid metal Ga-Sn alloy based ferrofluids with amorphous nano-sized Fe-Co-B magnetic particles. J. Mater. Sci..

[30] Yang C., Liu Z., Yu M., Bian X. (2021). Magnetic nanofluid based on amorphous Fe-Ni-B@OA particles applied in the treatment of oil slick. Soft Mater..

[31] Li Z.K., Li D.C., Chen Y.B. (2020). Study on the yielding behaviors of ferrofluids: A very shear thinning phenomenon. Soft Matter.

[32] Sudha J.D., Sivakala S., Patel K., Nair P.R. (2010). Development of electromagnetic shielding materials from the conductive blendsof polyaniline and polyaniline-clay nanocomposite-EVA: Preparation and properties. Compos. Part A.

[33] Ibiyemi A.A., Yusuf G.T., Olusola A. (2021). Influence of temperature and magnetic field on rheological behavior of ultra-sonicated and oleic acid coated cobalt ferrite ferrofluid. Phys. Scr..

[34] Vinod S., Camp P.J., Philip J. (2020). Observation of soft glassy behavior in a magnetic colloid exposed to an external magnetic field. Soft Matter.

[35] Basheed G.A., Jain K., Pathak S., Pant R.P. (2017). Dipolar interaction and magneto-viscoelasticity in nanomagnetic fluid. J. Nanosci. Nanotechnol..

